# Analysis of Rail Passenger Flow in a Rail Station Concourse Prior to and During the COVID-19 Pandemic Using Event-Based Simulation Models and Scenarios

**DOI:** 10.1007/s40864-022-00167-w

**Published:** 2022-05-13

**Authors:** Jeremy Lee, Marin Marinov

**Affiliations:** 1 EPS, Aston University, Birmingham, UK; 2 ESSCM, Aston University, Birmingham, UK

**Keywords:** Passenger behaviour, COVID-19 pandemic, Railway passenger station, Concourse, Ticket facilities, Event-based simulation models, Scenarios, Crowd management

## Abstract

During COVID-19, certain means were proposed to improve crowd management in the Birmingham New Street railway station. To validate the current system of crowd management in the station, this paper examines the rail passenger flow in the concourse of the Birmingham New Street railway station and the passenger interactions and queueing phenomena associated with it, mainly at the ticket machines, offices and gates, prior to and during the implementation of COVID-19 measures. The passenger behaviour in the concourse of the station was simulated using the SIMUL8 event-based simulation modelling package. Three different scenarios were modelled to analyse the changes and impacts from pre-COVID-19 and within the COVID-19 context. The results revealed that passenger behaviour in railway stations is changing due to COVID-19. Specifically, passengers are more likely to buy tickets using their smartphones or online prior to or whilst entering the station so that they can go through the station concourse with minimal queuing times and avoid contact with a facility of common use at the station, whereas those without tickets are more likely to be in a queue to buy their tickets in the station. For pre-COVID, the results showed that even with a reduced number of ticket machines, overcrowding inside the station was unlikely to occur, as 80% of all passengers in the simulation completed service within a 15-minute time frame. However, during implementation of COVID-19 measures, as the number of passengers using the station dropped significantly and more passengers bought their tickets using their smartphones and/or online, queueing times were also shorter, and thus passengers spent less time in the system. The simulation results were in accordance with the expected practice; hence the effectiveness of the simulation model was verified. Overall, as a result of this study, the following suggestions to improve crowd management in a railway passenger station concourse are proposed: encourage passengers to purchase tickets on their smartphones, remove ticket gates and replace them with sensors, and provide a one-way passenger flow system in the main concourse of the station.

## Introduction

### Background and Motivation

With the unprecedented lockdown in the UK during March 2020, the UK government instructed the public to stay at home and to follow lockdown restrictions. The restrictions included encouraging people to work at home and to avoid using public transport where possible. Consequently, this led to the number of rail passenger journeys dropping to historically low levels. As the Office of Rail and Road [22] reported, during the first quarter of 2020/2021, the rail passenger journeys in Great Britain fell to 35 million (8.1% of the 439 million in the first quarter of 2019/2020), which was the lowest recorded since the mid-nineteenth century. This indicates that many passengers are less confident and more uncomfortable with using public transport. A survey conducted by the Department for Transport [5] highlights that a large number of people have changed their travel behaviour, which has resulted in less travel by public transport and more by active travel, i.e. walking and cycling. The statistics from the same survey revealed that 88% of passengers did not get on a train during lockdown.

As train operating companies generate most of their revenue from rail passenger journeys, the COVID-19 pandemic has led to substantial economic consequences in the railway industry from decreasing numbers of rail passenger journeys, leading to operating costs exceeding the operating revenue from passengers. Thus, train operating companies are more likely to seek support from the government. For example, during the COVID-19 pandemic, Transport for London agreed to a second funding package from the government of £1.7 billion to support the transport services and network across London, until March 2021, to compensate for the reductions in revenue from passenger journeys [6]. However, without support from the government, train operating companies would need to reduce their operating costs by reducing the number of services and staff workers to ensure they survive and continue to operate.

Railway passenger stations, especially in urban areas, need to have an effective railway station concourse, as well as good ticket facilities for passengers to use. Looking at the railway statistics in terms of its passenger numbers in the UK (from 2019), according to a report produced by the Department for Transport [8], there has been an increase in passenger demand across all cities in the peak hours, including an increase of 2.4% in the morning arrivals and 1.2% in the evening departures, when compared to 2018. Also, observing the annual rail passenger journeys in 2018/2019, the data indicate that the West Midlands region has seen the highest growth of rail usage, to 101 million (up by 274%), since 1997/1998.

Furthermore, within the report, the majority of passenger rail trips are made by commuters (54%) for work and education purposes, whereas leisure and business purposes made up 31% and 10% of rail trips, respectively. However, a comparison using the data from the National Rail Passenger Survey produced by Transport Focus [28] revealed that commuters are the least likely passenger group to be satisfied with their overall journey, from station to train facilities, when compared to leisure and business passengers. The overall satisfaction with the journey for commuters was 75%, whilst ratings by leisure and business passengers were 84% and 88%, respectively.

Thus, the rise in rail passenger journeys, especially in the West Midlands, and the risks of overcrowding in a railway station can lead to detrimental impacts on passenger satisfaction ratings and, more importantly, the safety of passengers and others when using the station. Hence, there are opportunities to improve crowd movement along with making changes to the ticket facilities to help reduce queues. Also, during the COVID-19 pandemic, means such as a one-way system may be used to improve crowd management, which would allow passengers to move around the station concourse more safely and in an orderly way.

Since the redevelopment of Birmingham New Street station in September 2015, Network Rail [18] states that the size of the concourse is three and a half times larger than the previous concourse. This provides a greater capacity for passengers to use the station, which allows crowds to move around freely and safely. The redevelopment of the station has been justified, as in the estimates of station usage in 2019–2020, Birmingham New Street station was ranked as the fifth busiest station in Great Britain and the first outside London of 46,510,526 entries and exits [21].

Therefore, this study has selected the Birmingham New Street station railway station in the West Midlands to examine passenger behaviour in using the station concourse prior to and during COVID 19, with the purpose of capturing apparent changes, as the practice suggests that passengers are more likely to buy tickets using their smartphones or online in order to avoid contact with a facility of common use at a station. By evaluating this behaviour, better decisions can be made for equipping rail passenger stations in the near future and suggesting a set of means to improve crowd management.

### Aim and Objective

The aim of this study is to analyse the rail passenger behaviour in the Birmingham New Street station concourse, pre COVID-19 and during COVID-19, with a particular focus on the number of passengers entering the station and using its ticket facilities in the station concourse. Specifically, this study examines the passenger interactions and queueing phenomena, mainly at the ticket machines, offices and gates.

It is expected that during COVID-19, certain means would be implemented to improve crowd management.

Therefore, the objective is to conduct a study with reliable and informative sources including statistics for Birmingham New Street station, which will be useful for obtaining data from passengers, in order to construct simulation models using SIMUL8. The simulation models will be used to validate the current system of crowd management and passenger behaviour in the station, and to create and propose scenarios to examine different situations, such as the COVID-19 impacts in the number of passengers using the station.

### Paper  Organisation

The rest of the paper is organised as follows: Section 2 highlights the state of the art, which focuses on the areas around crowd management, passenger behaviour and the impacts of the COVID-19 pandemic on rail passengers and services. Section 3 explains the methods and rationale of conducting the field research. Section 4 focuses on examining the railway station concourse, platforms and its ticket facilities in Birmingham New Street station. Section 5 relates to setting up simulation models to validate and examine the system of crowd management in the station. Section 6 proposes three distinct scenarios in the simulation model. Section 7 presents and evaluates the data and results gathered from the simulation models, followed by suggestions for better crowd management in a station concourse. Section 8 provides the conclusions of the study, and lastly, Section 9 suggests possible future work on this study.

## State of the Art

### Crowd Management and Passenger Behaviour

In the case studies investigated in this section, many have stated that crowd management is a necessity and is more apparent in urban railway stations where queues and bottlenecks form in parts of the station.

In the first case study concerning crowd management and infrastructure design in railway stations, Kabalan et al. [10] highlight that with the increased urbanisation and population in cities, railway stations face two main issues of passenger comfort and safety. This suggests that crowd management needs to be carefully monitored, particularly in stations of high passenger flows. The authors defined crowd management as ‘to plan and execute the orderly movement of a crowd through infrastructure’ (Kabalan et al. 2017, p. 713). Hence, they created a framework to identify the density of crowds and passenger flow and to gather data on all aspects and functionalities of the station, in order to analyse the effectiveness of crowd management in the station. The data collection they planned is through the use of real- and non-real-time data, whilst using a combination of automatic and manual methods to carry out the research. Their conclusions revealed that there are not many studies of crowd management in railway stations; however, they recommend monitoring of crowd congestion to identify the patterns and causes of queue formation. Therefore, there is a potential for future work of this study to validate its framework by conducting field research and collecting data from a selected railway station.

Similarly, in another case study, Martella et al. [14] note that crowd management, with the help of technology, can reduce the volume of accidents in crowded events as well as ensuring the crowd’s welfare. Their definition of crowd management indicates that crowd management is divided into two parts. They suggest that 90% of crowd management is done through planning and preparations before the event, whereas the remaining 10% of the effort is the execution of the event itself. From their research, they provided several factors to look for during the planning stages of crowd management. This includes knowledge of the visitors, the location, managing the clients, cooperating with various institutions, organising crowd managers and staff, event type, and finally, preparing for the weather. These crowd management factors can be applied to events such as music festivals, sporting events and other large organised events. However, this could be applied to public transport stations, particularly in railway stations where large crowds could exceed the capacity of a station. This could lead to severe consequences of accidents and fatalities from overcrowding if crowd management is not well planned and executed during the event. Therefore, from their framework, they concluded that communications between crowd managers and the crowd need to be further improved upon. This can be achieved through greater use of technology in crowd management, to allow crowd movement to be managed more efficiently and safely.

However, even if crowd management is well maintained, Samson et al. [26] noted that overcrowding in metro stations, especially during peak hours, with a minimal number of ticket booths and turnstiles would lead to formation of long queues. This means that passengers would take longer to access the platforms, hence affecting the quality of the service. From their research, to highlight the problems and to propose a better strategy for handling high volumes of crowds, they developed a crowd dynamics model to analyse the movements and behaviour of crowds in the station. According to their results, the average turnaround time for single-journey commuters to get to the turnstiles from the entrance was 4 minutes during non-peak hours and 6 minutes during peak hours. On the other hand, this is dependent on the number of ticket booths and turnstiles, as well as other factors such as the layout of the station which they could have considered. Nevertheless, they concluded that to minimise overcrowding, their results suggest that at least seven ticket booths and five turnstiles are required. For non-peak hours, they suggested that metro stations should contain at least 10 ticket booths and five turnstiles to handle between 100 and 5000 passengers. Conversely, for peak hours, they recommended that metro stations which handle between 4000 and 7500 passengers should have at least 11 ticket booths and six turnstiles.

### Rail Passengers, Services and Operators During the COVID-19 Pandemic

The effects of the COVID-19 pandemic on rail passengers and services have undoubtedly led to changes since March 2020. Vickerman [29] highlights the concern regarding reduced ridership since the beginning of the COVID-19 pandemic and the requirement for passengers to maintain social distancing in public transport. The issue of reduced number of rail passengers means that railway operators would experience a decrease in revenue from ticket sales and some services being cut. Also, this would lead railway stations to reduce their ticket facilities at stations and provide more space for passengers to social distance, and to reduce their operating costs by reducing the number of staff. Thus, the UK government has taken action to provide financial support to the railway industry to prevent rail operators from becoming insolvent and allowing them to continue their services. Therefore, the government regards rail services as essential, particularly for key workers who require travel.

However, despite the financial support from the government on public transport, Rothengatter et al. [25] indicate that commuter and long-distance rail transport have been severely impacted by the COVID-19 pandemic. This is because cities with mass rapid transit, i.e. New York, London, Paris and Tokyo, are most impacted in terms of the decrease in the number of rail passengers making journeys, mainly for commuting. Also, rail passengers for long-distance rail transport, typically for leisure purposes, have been in decline since the start of the pandemic in March 2020. Thus, many railway operators experienced passenger and financial losses throughout 2020. On the other hand, this study has predicted and developed two scenarios of post-COVID-19: the first scenario, called ‘roaring 2020s’, which takes a historical comparison with the Spanish flu pandemic during 1918, and the second, called ‘thoughtful 2020s’, which predicts and proposes substantial changes in the transport sector as a result of human behaviour, political attitudes, environment and economic influences.

With the ongoing effects of the COVID-19 pandemic, long-term impacts would include an observable shift from public to private transport mode. As Das et al. [4] suggest, there has been a change in passenger behaviour in using public transport when, during COVID-19 lockdowns and restrictions, the UK government has discouraged passengers from using public transport. Also, there are measures in place for public transport, such as reduced passenger capacity in railway stations and carriages to allow passengers to maintain social distancing. Thus, this would lead passengers to switch modes of transport, for example, from commuting by train to their own car. Therefore, this study found that passengers are likely to avoid public transport throughout and after the pandemic, as the findings showed that 80% of car commuters would continue to travel by privately owned vehicles through post-COVID-19, including a share of 18% from public transport users.

### Summary of the State of the Art

The first two case studies of crowd management and passenger behaviour both developed a framework to examine the effectiveness of crowd management. The first one indicates that the analysis of crowd management, density of crowds, passenger flow and behaviour and other factors requires specific data to carry out the research, Kabalan et al. [10]. The other case study, by Martella et al. [14], highlights that the majority of crowd management efforts involve planning and preparation rather than the actual execution of an action. This means that in the design of a new railway station, ticket facilities such as the number of ticket gates and ticket machines need to be considered to ensure there is enough capacity for passengers inside the station, with minimal crowding and queuing. However, despite both studies lacking actual results, the framework would help to focus on key elements which would be fundamental for this research paper in developing simulation models. This is where the third case study by Samson et al. [26] is relevant to this paper, because they developed a crowd dynamics model which analyses the movements and behaviour of crowds in the station. This can then be compared with the results of the current study, which would be useful in evaluating the effectiveness of crowd management in a railway station.

Conversely, taking into consideration the impacts of the COVID-19 pandemic, all three studies have highlighted that the demand of passengers using public transport have reduced. Also, the studies by both Vickerman [29] and Rothengatter et al. [25] express some concerns with transport services, especially for rail services. This is because railway operators have had to reduce their services and request financial support from the UK government to survive in the industry due to reduced operating revenue from passengers. Furthermore, in the post-COVID-19 scenario, Das et al. [4] anticipated that there has been a change in passenger behaviour, from using public to private transport modes. This means that passengers are more likely to switch to their own cars than use public transport, as passengers may want to avoid using public transport, such as rail transport, where they would have to maintain social distancing and wear face coverings. Therefore, the COVID-19 pandemic will certainly have long-term effects on the railway industry, particularly on its passengers, services and operators.

## Data and Methodology

### Initial Research Methodology

To conduct and collect the data for the field research, the Birmingham New Street station, based in the West Midlands, was selected.

The method of field research in this case is to observe the pattern of crowd movements within the station, in accessing the platforms from the station entrance. Hence, throughout the crowd movements, it is necessary to examine the ticket facilities and their layout, i.e. ticket offices, ticket machines, ticket gates, platforms and the station concourse.

For collecting primary data, equipment and resources such as a stopwatch, measuring tape and notepad would be required to record the data. This would then be transferred into the simulation modelling, specifically the timings and size of crowds in the station, to be inputted as the variables in the system.

### Changes in the Research Methodology Due to COVID-19

However, with the COVID-19 restrictions during the conduct of this study, field research was deemed unfeasible due to safety concerns such as maintaining strict social distancing rules inside the station. Thus, it became necessary to use secondary data such as from the Office of Rail and Road and other sources to collect statistics. These statistics were used to calculate and make reasonable assumptions and estimates based on the existing data in order to provide variables in the simulation model.

## Examining the Railway Station Concourse, Platforms and Ticket Facilities

### Station Concourse and Platform Layout

For the station concourse itself, the main function is to provide enough room for passengers to move through and wait inside the station, Network Rail [17]. The concourse should also provide a clear layout to ensure passengers are able to get through the station easily and comfortably, which includes having clearer wayfinding signage, removing unnecessary obstacles, and well-managed station flooring, i.e. preventing slipping hazards and clearing litter from the ground, Network Rail [15].

**Fig. 1:**
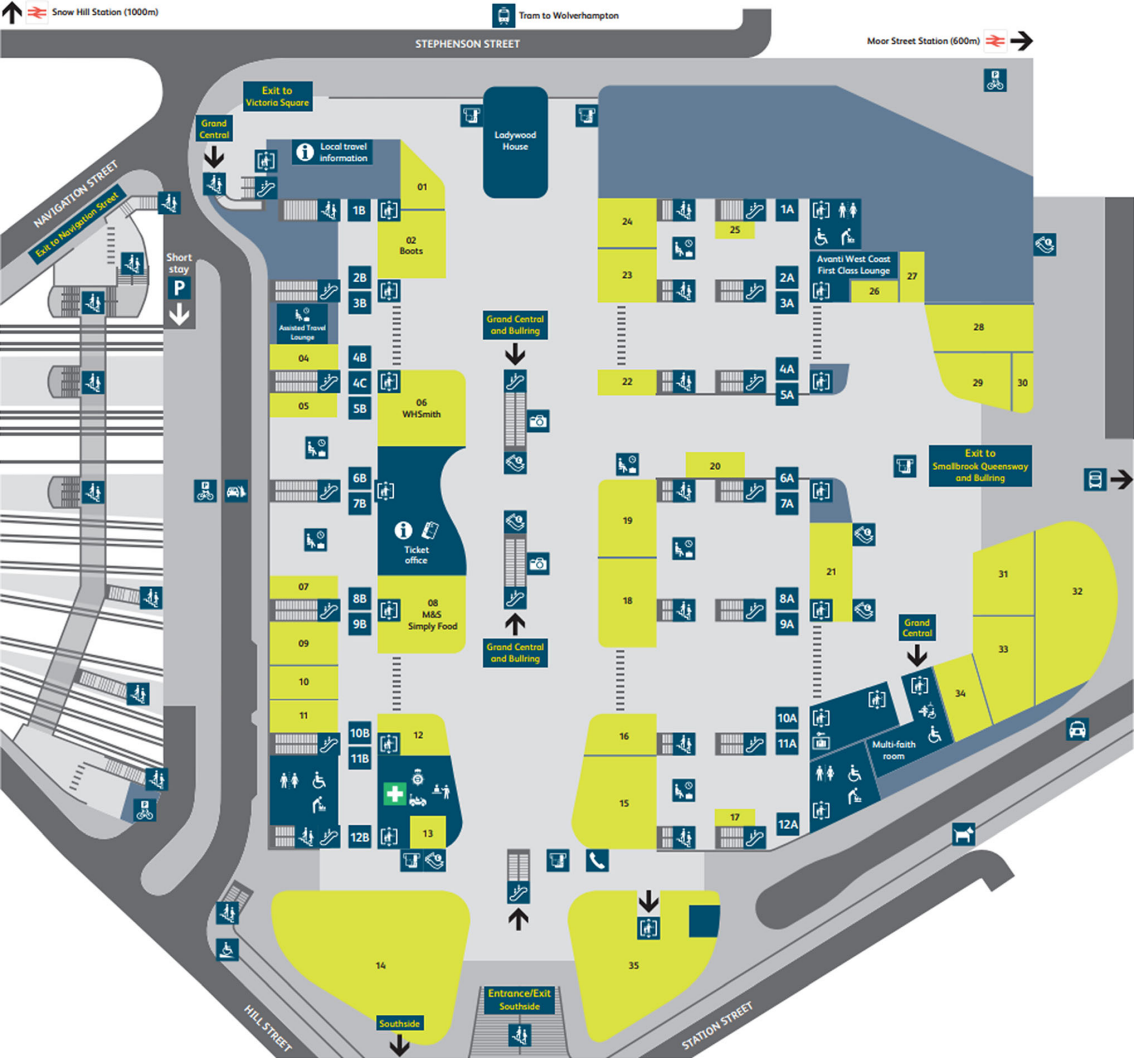
Birmingham New Street station design layout, Network Rail [19]

For platform layout, as illustrated in Fig. 1, all 12 platforms are located below the main concourse and are accessible via staircases, lifts and escalators. However, with the complexity of the station, passengers accessing different platforms are required to go to one of three lounges which are separated as Blue, Red and Yellow Lounges. Each lounge is separated by ticket gates from the concourse, meaning that passengers are required to have valid train tickets to access the platforms. The Red Lounge is accessible to all 12 platforms (platforms 1B–12B and 4C), whereas the platforms for the other two lounges are separated, where the Blue Lounge provides access to platforms 1A–5A, and the Yellow Lounge provides access to platforms 6A–12A.

Examining the entry and exit points of the station, it has six main entrances/exits, including two entrances/exits connecting towards the shopping centre of Grand Central & Bullring, along with an exit only to Navigation Street in a separate concourse which is connected to all platforms.

### Ticket Facilities

#### Ticket Gates

For the ticket gate layout of the station, there are 62 ticket gates in total. The ticket gates are distributed into different areas of the station to allow multiple entrances and exits for passengers, as shown in Table 1.

**Table 1:** Ticket gates in lounges/areas of Birmingham New Street station

Entrance/exit points	Station lounge/concourse areas	Connected platforms	No. of ticket gates
Stephenson Street	Red Lounge	1B–12B and 4C	11
Southside and Hill Street	Red Lounge	1B–12B and 4C	10
Stephenson Street	Blue Lounge	1A–5A	11
Smallbrook Queensway and Bullring	Blue Lounge	1A–5A	6
Southside and Hill Street	Yellow Lounge	6A–12A	10
Smallbrook Queensway & Bullring	Yellow Lounge	6A–12A	6
Navigation Street	Exit-only concourse	1–12	8
		Total	62

#### Ticket Offices

Also, for passengers needing to purchase train tickets, the ticket office is located in the centre of the main concourse, along with the ticket machines next to the ticket office. The ticket office itself contains 12 counters for passengers to purchase tickets, by either cash or card, and ask for other ticketing advice. However, depending on the time of day and other circumstances, typically not all counters are open, which could be due to the reduction in the number of staff in order to reduce station operating costs.

#### Ticket Machines

Hence, there are ticket machines in the main concourse which provides 15 ticket machines (see Table 2 for more detailed ticket machine payment methods), where passengers can purchase or collect their tickets from the machines.

**Table 2:** Ticket machine payment methods in the main ticketing area of Birmingham New Street station

Ticket machine payment methods	No. of ticket machines
Card only	7
Cash and card	8
Total	15

#### Comparing Ticket Office and Ticket Machine Use

Ticket offices in stations are deemed necessary for some passengers to ensure they purchase the correct rail ticket, with the assistance of ticket office staff. However, using new technologies, there is a proposal for the future removal of ticket gates and ticket offices to provide additional capacity in the station to improve the customer experience, as well as reducing operating costs in the station, Network Rail [16].

With the growth and advancements in technology, ticket (vending) machines have become more dominant than ticket offices for most passengers when purchasing rail tickets quickly. The Office of Rail and Road [20] conducted research to examine passengers’ ability, using ticket machines, to make the most appropriate rail ticket purchase. This was done through a mystery shopping exercise which involved mystery shoppers who purchased a range of rail ticket types at ticket machines. Their results revealed that, from their total sample size of 739, 91% of shoppers selected the most appropriate ticket, and the majority (75%) were overall satisfied in using the ticket machines to purchase tickets. However, notably, the report revealed that 89% of shoppers using ticket machines did not provide information, showing other ticket types which may have displayed from the ticket office.

Hence, not all passengers are purchasing their tickets from ticket machines, as some passengers are concerned they may purchase a wrong type of ticket which may lead to them being overcharged or at risk of incurring a penalty fare, i.e. travelling during on-/off-peak hours when the ticket is not valid during those hours. Therefore, some passengers are likely to purchase their tickets through a ticket office or online, which allows passengers to buy a ticket in advance on their smartphones to go straight through to the ticket gates, thus reducing queuing time.

## Simulation Modelling Using SIMUL8

### SIMUL8 and Considerations Before Building the Simulation Model

The use of the SIMUL8 software modelling package for rail operations and management-focused research has been demonstrated in various studies [3, 12, 13, 23, 24, 31]. The software allows for both analysis of the queuing system and examination of the passenger behaviour in interactions with ticket facilities. Hence, the simulation model discussed in this paper was designed to consider any passengers who needed to buy valid train tickets from either ticket machines or a ticket office to pass through the ticket gates, as well as those who had already purchased their tickets beforehand (usually as advance tickets) and could go straight to the ticket gates.

For the simulation model itself, three scenarios are observed by first considering the current system of crowd management with all working ticket machines before the COVID-19 impacts on passenger entries, the second being the reduction in working ticket machines whilst retaining the same settings as the first scenario, and finally examining the impacts of COVID-19 in passenger entries to analyse the difference in passenger movements and interactions (see Table 3).

**Table 3:** Scenario types to be simulated

Scenario no.	Scenario description
0	Pre-COVID-19 passenger entries
1	Reduction in the number of ticket machines
2	COVID-19 impacts on passenger entries

However, there are some limitations when using SIMUL8, as the simulation software is optimised for and can more easily recreate a linear one-way flow system than a two-way flow system, to ensure the model functions correctly. Also, since this simulation model is intended to focus on the queue sizes and passenger behaviour, a large amount of data is to be simulated in the model. However, the SIMUL8 software package used for this study is limited in working with excess amounts of data; for instance, having more than 10,000 entities as a working activity in the simulation model would likely cause the software to freeze and to stop working.

Hence, to avoid the risk of the software becoming unstable, for the simplicity of simulation modelling, this model focuses only on the passenger departures in Birmingham New Street station between where passengers enter the station (start of simulation) and reach the platform levels (at the end of simulation).

Also, since Birmingham New Street station has multiple lounge/concourse areas, this simulation model focuses only on the Red Lounge area, where passengers are able to access all 12 platforms.

The simulation model runs for 5 hours between 14:00 and 19:00, which covers the evening peak hours where there are more passenger departures than arrivals, especially in urban railway stations. Additionally, the simulation model conducts trials five times to ensure further accuracy of the results.

Thus, this simulation model aims to focus on a smaller scale of passenger departures/entities, rather than the whole day and the whole station, to ensure model simplicity and precision.

### Assumptions from the Data Collection for the Simulation Model

Since, as mentioned in Section 3.2, field research could not be done due to COVID-19 restrictions, alternative methods in gathering data were needed, such as the use of secondary data. Thus, the data are based on the number of passenger entries through the ticket gates in the station. These data are also used to calibrate and validate the simulation model.

However, before using the secondary data as variables for the simulation model, assumptions and estimates needed to be calculated. Hence, using the Office of Rail and Road [21] statistics, knowing that there were 46,510,526 total passenger entries and exits in Birmingham New Street station in 2019–2020, assumptions were made to calculate the daily number of passenger entries. This was calculated by halving the total annual passenger entries and exits in Birmingham New Street station, to focus only on the passenger entries, which is 46,510,526 × 0.5 = 23,255,263 entries. This figure was used to calculate the daily number of passenger entries, which is 23,255,263/365 = 63,713 entries per day (rounded to nearest whole number). This figure was calculated for the simulation model, as it provided an estimate of how many passengers entered through the station ticket gates per day. This was to ensure validation of input in the simulation model.

For further validation of the simulation model, it was important to calibrate the model against the number of departures throughout a whole day during every hour. Using the statistics from a spreadsheet in the Rail Table, DfT [7], an hourly time band in the number of passenger departures from the city centre was used to validate the output of the simulation. These statistics were converted into percentages to determine the number of passenger entries during a specific time band as a percentage of the whole day. Hence, using the estimated 63,713 entries per day in Birmingham New Street station, using Table 4, the combined percentage of passenger departures between 14:00 and 19:00 constituted about 51%.

**Table 4:** Number of departures by rail in Birmingham City Centre stations in 2019, by time band (adapted from Dft [7])

Time band	Passenger departures (%)
Start of service to 06:59	2
07:00–07:59	5
08:00–08:59	7
09:00–09:59	4
10:00–10:59	4
11:00–11:59	4
12:00–12:59	4
13:00–13:59	5
14:00–14:59	6
15:00–15:59	8
16:00–16:59	12
17:00–17:59	16
18:00–18:59	9
19:00–19:59	6
20:00–20:59	4
21:00–21:59	2
22:00–22:59	2
23:00 to end of service	1
Total	100

Thus, the estimated number of passenger entries for this simulation model would be 63,713 x 0.51 = 32,494 entries between 14:00 and 19:00 (rounded to nearest whole number). However, this estimated figure takes into consideration all other passengers entering through the ticket gates to all lounge areas. Therefore, for the purpose of this model, this estimated figure in theory would be lower when looking at the passengers entering the Red Lounge area.

### Building the Simulation Model Using SIMUL8’s Building Blocks

An essential step in creating the simulation model to analyse the effectiveness of crowd management in Birmingham New Street station is the use of the building blocks from SIMUL8 to create a functional simulation model.

**Fig. 2:**
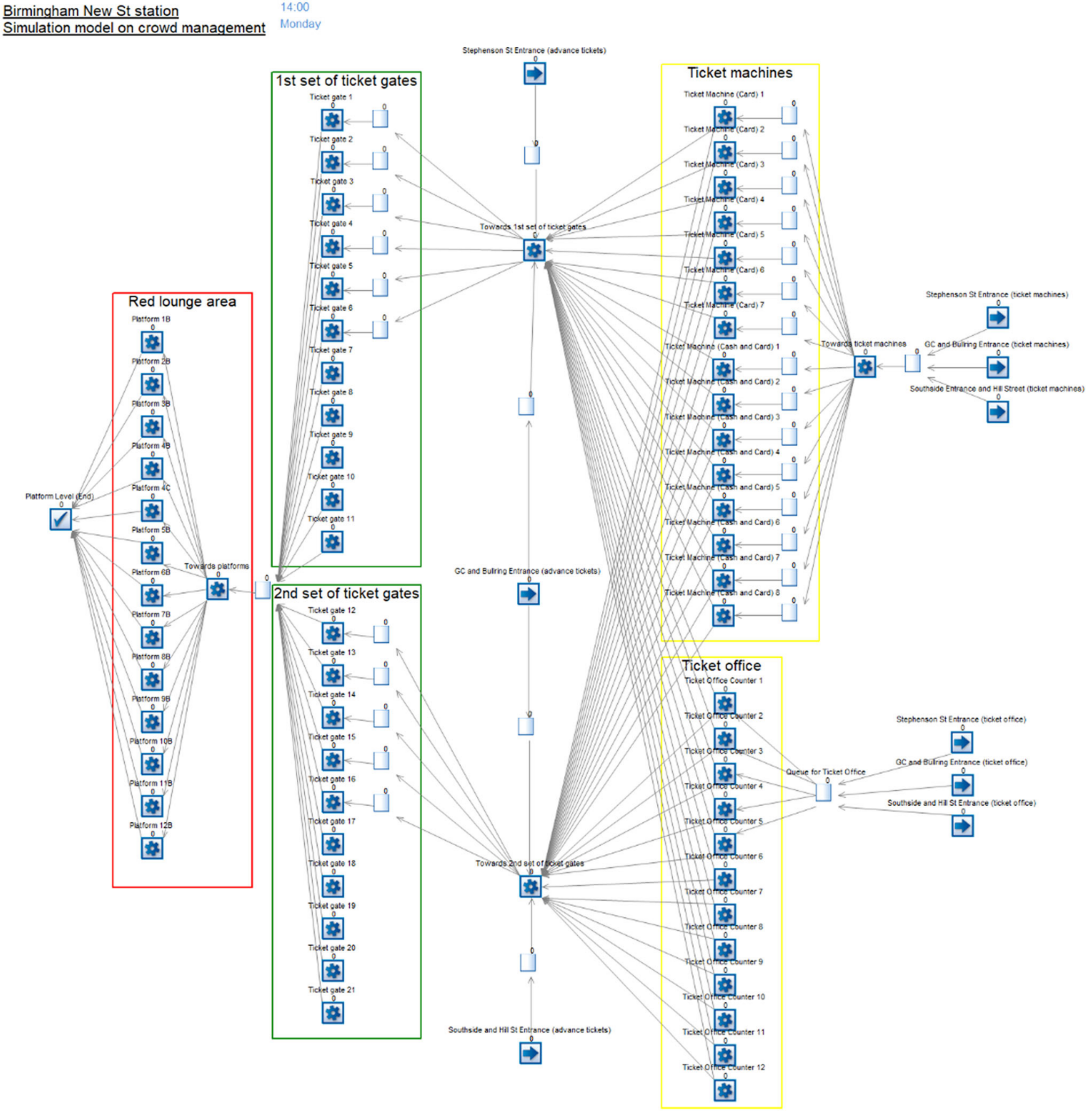
Simulation model of crowd movements to analyse the effectiveness of crowd management, using SIMUL8’s building blocks

For this simulation, four building blocks are used throughout the model (see Fig. 2):

1. *Start point* Acts as an arrival of passengers entering the station. The start point can have multiple entry points for passengers to arrive at the station.
2. *Queue* This point enables passengers to wait for the next activity.
3. *Activity point* The activity serves multiple proposes by either a machine or a human activity, i.e. ticket machines, ticket office counters, ticket gates/barriers and escalators to the platform level.
4. *End point* Serves as the completion point for passengers in the simulation model.

**Fig. 3:**
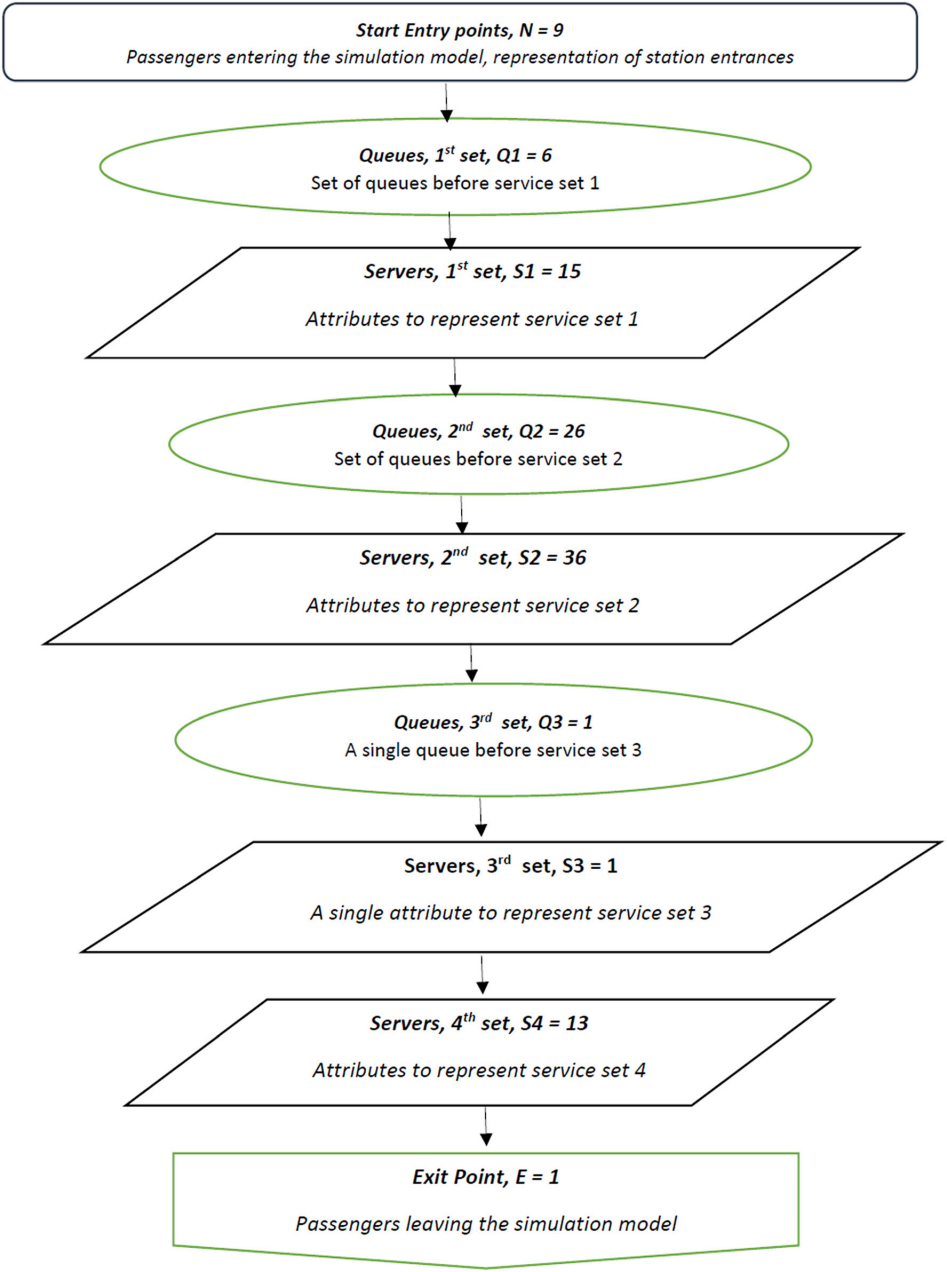
Stages of simulation model development

A flow chart of the simulation model illustrating the stages of model development, implemented using SIMUL8, is shown in Fig. 3.

### Labels

**Fig. 4:**
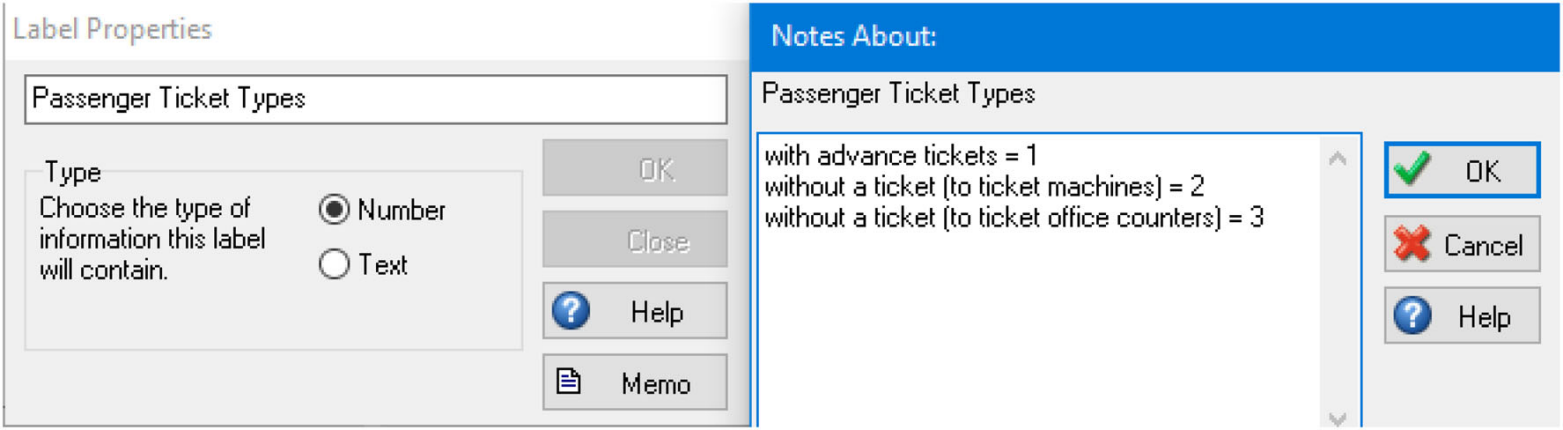
Labelling of ticket passenger types

Since this simulation model is intended to focus on the entry of passengers who already have tickets and those without tickets (would have to go to either a ticket machine or ticket office), labels are used to segregate the results at the end point to make it easier to compare the results. Also, the labels can be used at the start point to assign different ticket passenger types. These are allocated as label values in SIMUL8 as shown in Fig. 4.

### Start Point Properties

**Fig. 5:**
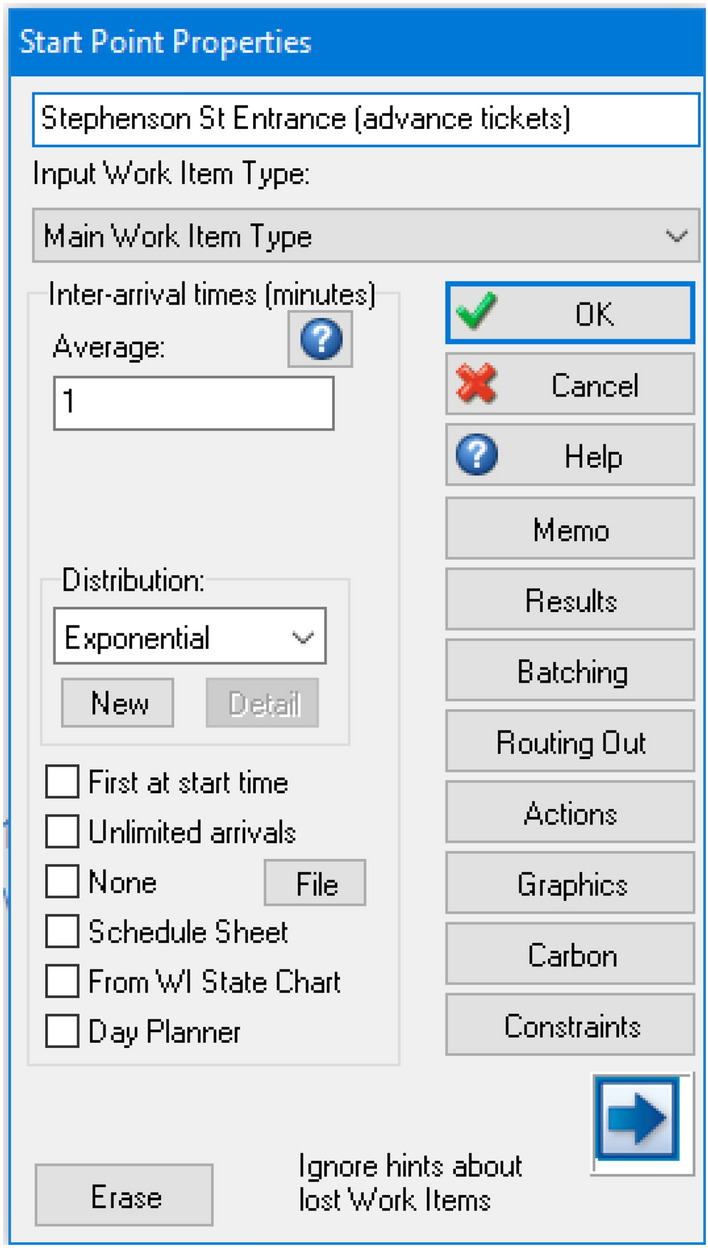
Start point properties for the Stephenson St entrance (advance tickets)

In each of the start points, certain properties need to be inputted to ensure the system estimates the appropriate number of passengers entering the simulation model. These include inter-arrival times, batching and actions with the use of labels. See Fig. 5 as an example of start point properties.

For this simulation model, there are three different entrances for passengers to enter Birmingham New Street station. The three entrances in the station are Stephenson Street, Grand Central & Bullring, and Southside & Hill Street.

#### Inter-arrival times

Since there are three entrances in the model, some entrances may observe passengers coming into the station at a more regular occurrence. For all three inter-arrivals times, the distribution is set to be exponential because the rate of passengers entering the station is rather random as in reality; hence, setting the distribution to exponential provides the simulation model with some realism.

However, some entrances such as Stephenson Street provide access to the West Midlands Metro tram services which connect from other areas of Birmingham and West Midlands. This implies that there would be more passengers entering the station at a regular occurrence, as they would change to another mode of public transport acting as an interchange station. For the Grand Central & Bullring entrance to the station, it provides two sets of escalators for passengers to travel down into the station from the two shopping centres. In contrast with the third entrance, the Southside & Hill Street entrance provides access to taxis and several bus stops. The inter-arrival times for all three entrances in the model are shown in Table 5.

**Table 5:** Inter-arrival times of passengers at the selected entrances in Birmingham New Street station

Entrance points	Inter-arrival times (min)
Stephenson Street	1
Grand Central and Bullring	2
Southside and Hill Street	3

#### Batching

Batching in SIMUL8 allows for multiple passengers to enter the station at the same time, for instance, there may be groups of families entering the station. However, it is not possible to predict the exact number of passengers entering the station at one time. Hence, the distribution of batching is set at exponential for all start points to avoid partiality in the results. However, some entrances may be busier than others, as there are factors such as connections to shopping centres and business areas which may attract more passenger entries into the station. The batching sizes for all three entrances (also highlighting the variations of three ticket passenger types) are shown in Table 6.

**Table 6:** Batching in the number of ticket passenger types entering Birmingham New Street station

Entrance points	Batch sizes of passenger entries into the station		
	With advance tickets	Without tickets, towards ticket machines	Without tickets, towards ticket office counters
Stephenson Street	8	4	1
Grand Central and Bullring	6	3	1
Southside and Hill Street	4	2	1

The rationale for setting the batch size lower than the passengers with advance tickets is to prevent queues from becoming overwhelmingly blocked, especially those who need to purchase train tickets from either a ticket machine or ticket office. In reality, passengers are more likely to use ticket machines than a ticket office to purchase their train tickets. According to research on passengers’ experience of ticket machines conducted by the Office of Rail and Road [20], 93% of the mystery shoppers queued for less than 2 minutes, and 73% were able to complete their transaction at the ticket machines in less than 3 minutes. Hence, it is reasonable to assume that more passengers use the ticket machines than going to a ticket office, as it is generally quicker to purchase tickets on machines, thus providing a self-service.

#### Actions

**Fig. 6:**
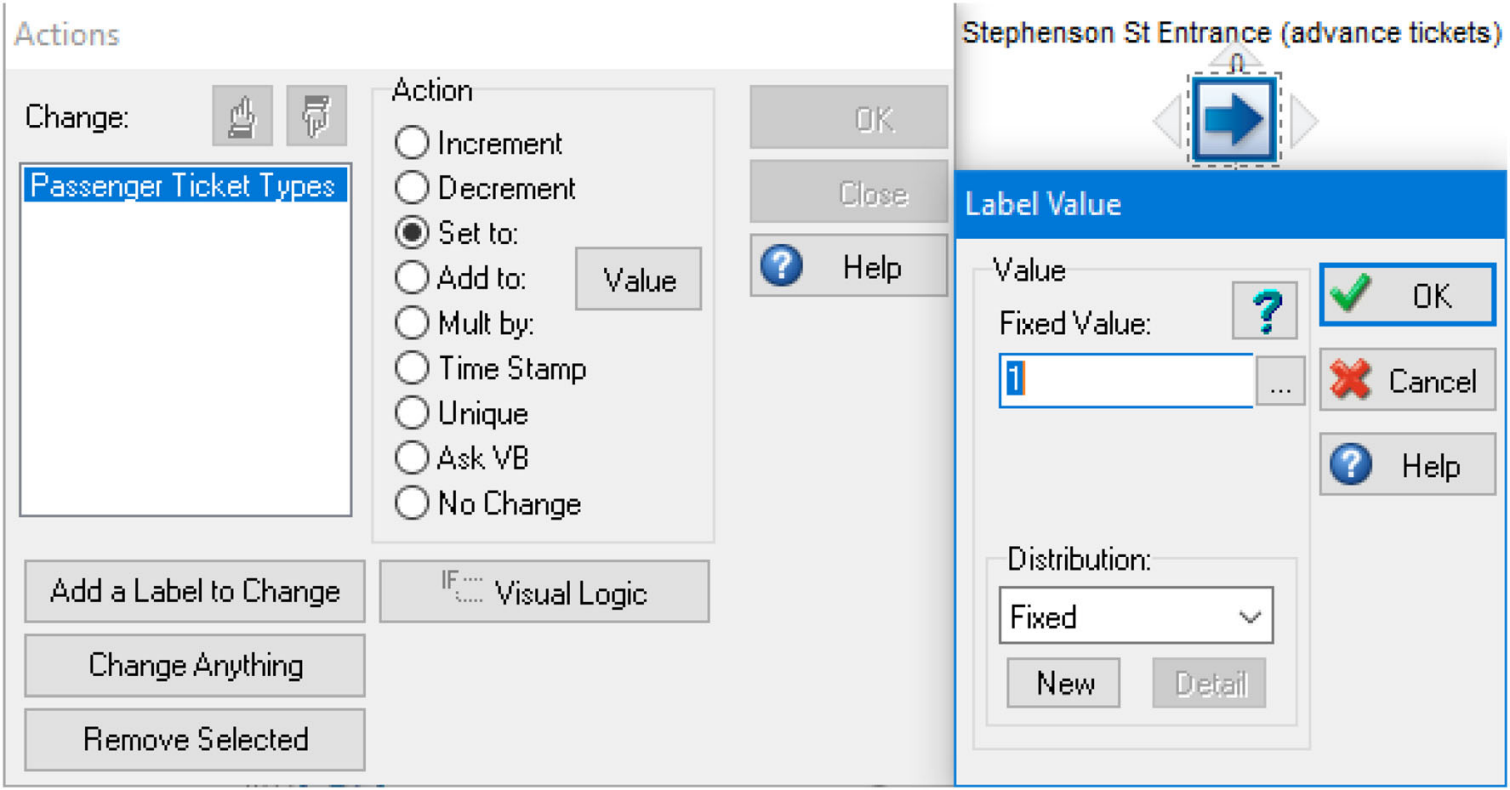
Configuring the label value for the start point of passengers with advance tickets within the actions setting

For all start points, actions were used to assign values to labels to provide segregated results at the end of the simulation. Labels need to be created beforehand (see Sect. 5.4 about labels) in order to assign different ticket passenger types for each value in the actions at each start point. The values given for the three ticket passenger types are shown in Table 7 and Fig. 6, as an example, for setting up the actions. All values are set as fixed, to ensure the correct label is set.

**Table 7:** Values to assign each label for the actions setting

Ticket passenger types at each start point	Value
With advance tickets	1
Without tickets (ticket machines)	2
Without tickets (ticket office)	3

### Activities, Routing Out and Queues

The activity points were used the most throughout the simulation model. These activities are utilised when passengers interact with them. For this model in particular, activities include ticket machines, the ticket office, ticket gates and escalators to platforms. Most activities have a distribution in the amount of time for passengers to interact with.

**Fig. 7:**
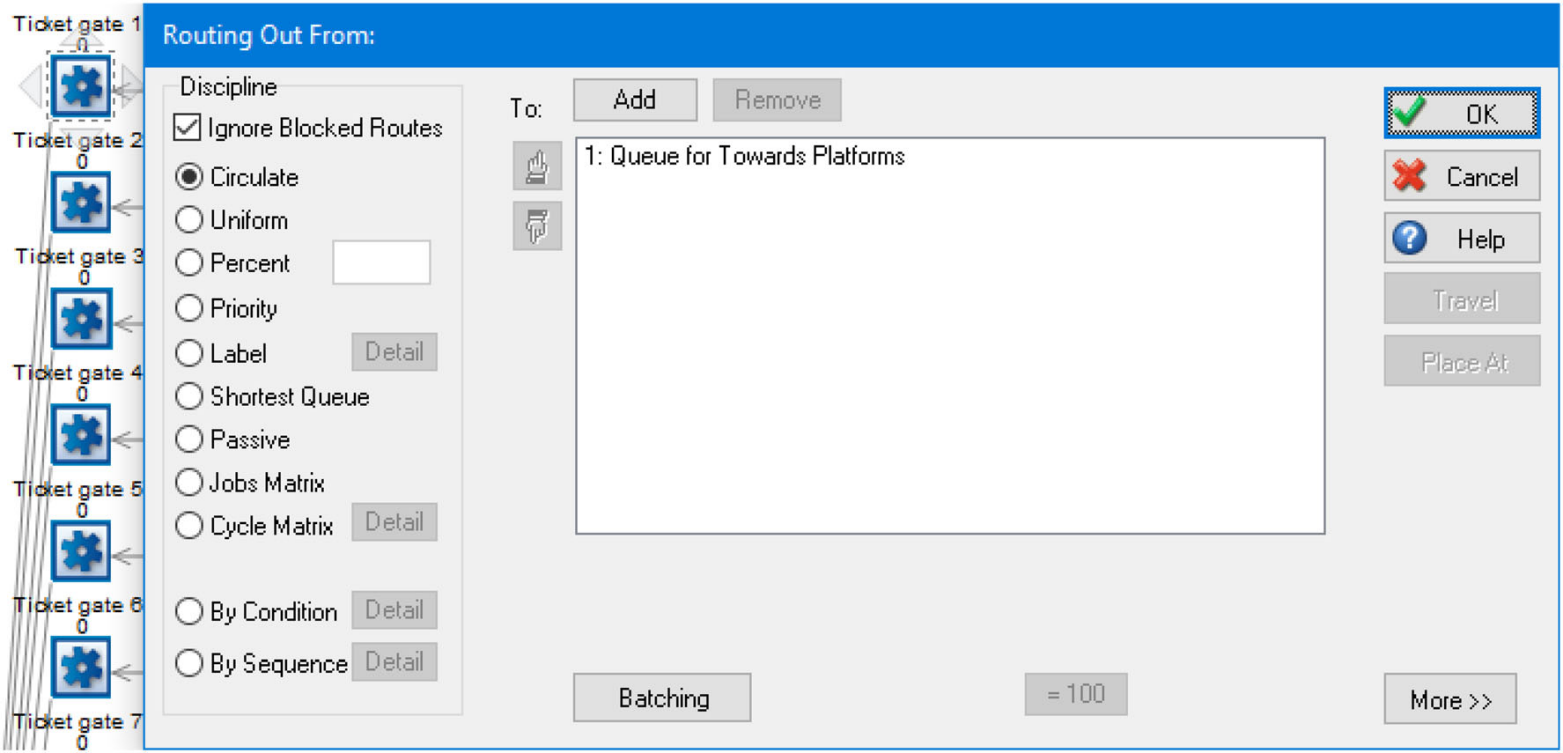
Routing out option for a ticket gate as an activity point

After they have finished an activity, they will move to the next activity; however, when there is more than one activity for the passenger to move from the previous activity, a routing out option will be used. The routing out options allow for the activities to allocate passengers to the next process using the routing rules (see Fig. 7 as an example).

For the queuing systems in the model, the queue points are laid out after each start and activity point (except at the end of the model). This ensures that the passengers are able to wait in the system where there is an activity in use at full capacity. Hence, the queuing system in this model provides useful data for the results at the end to analyse the effectiveness of crowd management.

#### Ticket Machines

There are 15 ticket machines in total for this model, which are all connected with each individual queue. Before the passengers start queuing for the ticket machines, using the routing out at the ‘towards ticket machines’ activity, the routing rule is set as ‘shortest queue’, meaning that passengers mostly go towards the ticket machines with the lowest number of passengers in the queue. The time distribution for all ticket machines is set as uniform to allow a random occurrence between the lower and upper time values. However, depending on the ticket machine payment methods, the time settings are different, as seen in Table 8.

**Table 8:** Uniform distribution showing the time values set for two types of ticket machines

Data points	Time values (min)	
	Card only	Cash and card
Lower	1	1
Upper	2	3

After the passengers have purchased their tickets, they will go to the next activity point. Since there are two activity points towards the two sets of ticket gates, routing out is used, and the rule is set as uniform. This means that there is an equal chance for passengers to go to either set of ticket gates.

#### Ticket Office

For the ticket office, it provides 12 ticket office counters for passengers to purchase tickets. However, for this model, the ticket office counters will be halved to six counters, which anticipates the decline in the use of ticket offices to reduce workforce expenses. Comparable to ticket machines, this uses the uniform time distribution which has lower and upper bounds of 2 and 4 minutes, respectively. This also uses the same routing rules, set as uniform. But unlike for ticket machines, there is a single queue at the ticket office, which connects with all counters. This means that the passengers will not have the choice of queueing up at an individual counter. Hence, this is likely to lead to a larger queue forming than each of the individual queues at the ticket machines.

#### Ticket Gates

Since there are 21 ticket gates in the Red Lounge area, separated into two sets of ticket gates, not all of them will be utilised, because this model focuses only on the passenger departures into the station. This means that half of the ticket gates will be used, as in reality there will be passengers departing and arriving at the station, using the ticket gates at both ends. In case there is an odd number, such as at the first set of ticket gates near the Stephenson Street entrance, containing 11 of them, six out of 11 ticket gates will be used in the model as it is simulated during the evening peak hours. In contrast, the other set of ticket gates will use five ticket gates, bringing the total to 11 to be utilised in this simulation model. The time distribution for all ticket gates is set at triangular, which allows for lower, upper and modal time values as seen in Table 9.

**Table 9:** Triangular distribution showing the time values set for ticket gates

Data points	Time values (min)
Lower	0.05
Upper	0.3
Mode	0.1

#### Escalators to Platforms

These last sets of activities at the end of the system, the platform numbers in the model denote escalators to platforms. When the passengers have arrived in the Red Lounge area, after passing through the ticket gate, the passengers will go towards a platform, meaning that the routing out setting needs to be used, since there are multiple activities for passengers to go from the previous one. Hence, the routing out rule is set as uniform to ensure there is an equal chance for passengers to go to any of the platforms, without any bias in the system. The time distribution for the passengers to travel down the escalators to the platforms is set at an average of 0.5 minutes. Also, the escalators can accommodate more than one passenger at a time, when compared with other activities such as individual ticket machines, which can only be utilised by one passenger at a time.

**Fig. 8:**
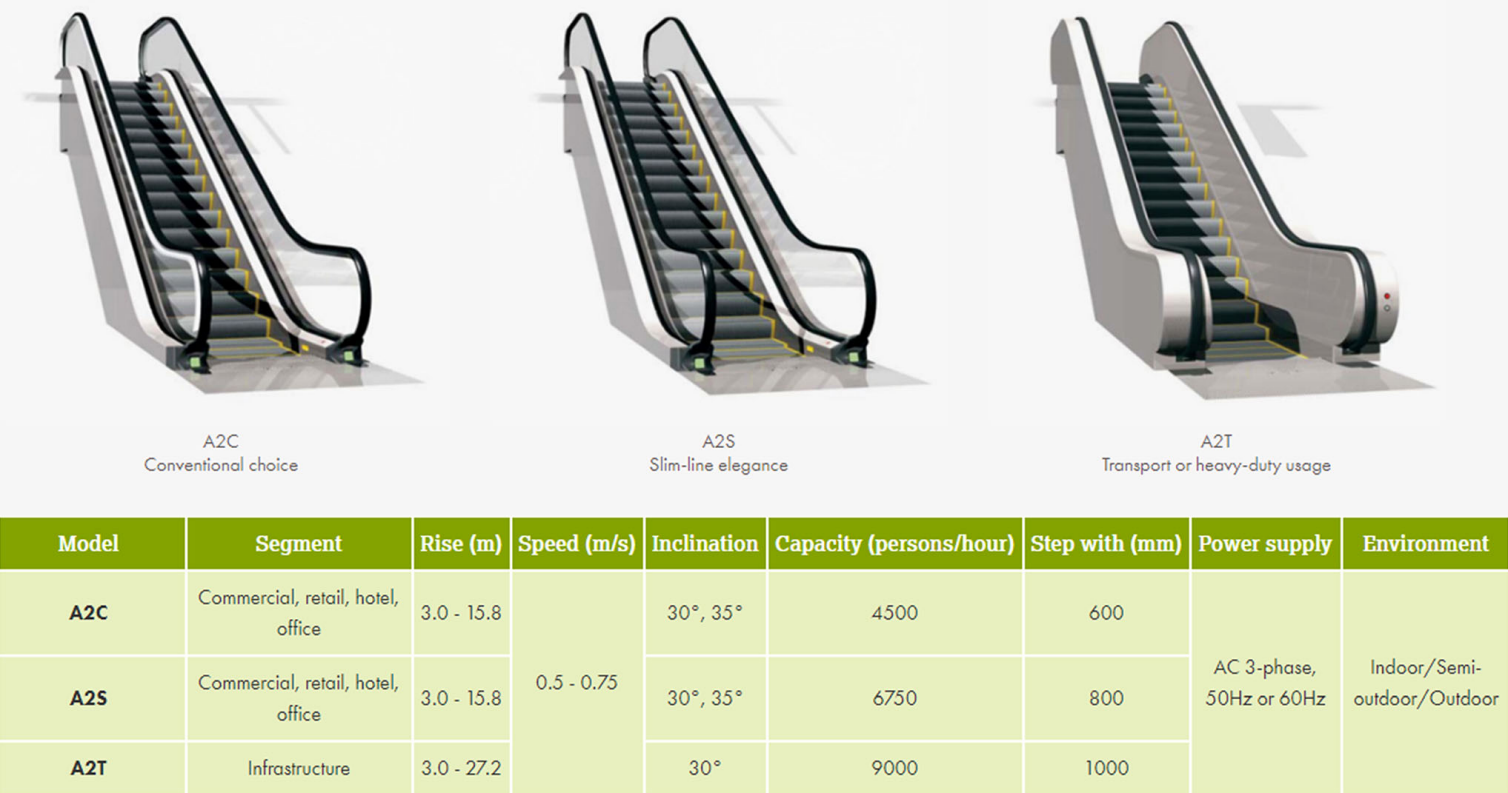
General specification for each escalator model, Stannah [27]

The escalator capacity for each one is estimated to be 9000/(60 × 2) = 75 persons per 30 seconds, as calculated using Stannah’s [27] specification of the A2T escalator model’s capacity of 9000 persons per hour (see Fig. 8). To allow the model to accommodate up to 75 passengers on the escalators, this is done simply by replicating the number of activities from the SIMUL8’s properties for escalators from 1 to 75 times.

## Modelling Scenarios

### Scenario 0

As discussed in Sect. 5, the framework for Scenario 0 is to create a simulation model to examine the typical crowd dynamics of their movements in Birmingham New Street station, to analyse the effectiveness of crowd management. The purpose of Scenario 0 is to validate the simulation model as close to reality before COVID-19. This is done using data from the Office of Rail and Road statistics for 2019/2020, and making reasonable assumptions and considerations for all activities involved, i.e. the number of passenger entries into the station and the number of ticket office counters to be used in the simulation.

### Scenario 1

As in reality, some ticket machines may need maintenance due to a fault in the machine itself. Hence, assuming that several ticket machines are out of service, this scenario imposes a reduction by one third in the total number of ticket machines, meaning that the 15 ticket machines in the system will be reduced to 10; see Table 10 for the reduced number of ticket machine payment methods.

**Table 10:** Reduced number of ticket machine payment methods: Scenario 1

Ticket machine payment methods	No. of ticket machines
Card only	5
Cash & card	5
Total	10

For validation, Scenario 1 uses the same model as Scenario 0, retaining the number of ticket office counters at six whilst reducing the total number of ticket machines to 10.

### Scenario 2

This scenario features the greatest change from the model used for Scenario 0, as it focuses on the impacts of the COVID-19 pandemic during the UK’s lockdown in March 2020 on the number of passengers using the station and the changes to station design elements. These changes are required to enable social distancing for passengers to ensure that everyone is able to get around safely through the station. Hence, there will be a limited number of ticket office counters, ticket machines and ticket gates, in order to provide more space for passengers to maintain social distancing.

Thus, for validation of Scenario 2, the same model from Scenario 0 is used, but with the changes in the number of passengers entering the station, as well as most activities (which interact with passengers) in the simulation to be reduced, meaning that the total number of ticket machines will be reduced from 15 to 8 (see Table 11), along with a reduction in ticket offices by two thirds, from six to two counters, and additionally a reduction in the number of ticket gates towards the Red Lounge area (see Table 12). This is to reflect the lower number of passengers entering the station, which also encourages passengers to have already purchased their tickets beforehand.

**Table 11:** Reduced number of ticket machine payment methods: Scenario 2

Ticket machine payment methods	No. of ticket machines
Card only	4
Cash and card	4
Total	8

**Table 12:** Comparing the number of ticket gates for both sets of ticket gates across three scenarios

Sets of ticket gates entering the Red Lounge area near the entrance/exit points	No. of ticket gates for Scenario 0 and 1	No. of ticket gates for Scenario 2
First set: Stephenson Street	6	3
Second set: Southside and Hill Street	5	3
Total	11	6

Hence, to determine the number of passengers entering the station during the COVID-19 lockdown in March 2020, knowing that there are only 8.1% of 439 million passengers (from the first quarter of 2019/2020) making journeys in the first quarter of 2020/2021, ORR [22], it is possible to calculate the number of passenger entries in Birmingham New Street station for Scenario 2, reflecting the impacts of COVID-19 lockdown in March 2020, by using the same number of passenger entries for Scenario 0, but reduced by 8.1%. Therefore, using the same time frame as Scenario 0, between 14:00 and 19:00, the estimated number of passenger entries in Scenario 2 will be 32,494 × 0.081 = 2632 entries (rounded to nearest whole number).

For the inter-arrival times between Scenarios 0, 1 and 2, all three entrances are increased by five times (see Table 13), whereas the batching sizes for all entrances have decreased variably (see Table 14). The rationale for this is to ensure the appropriate number of passengers entering the station in the simulation model, to reflect the decrease in passengers entering the station and using its ticket facilities due to COVID-19. Thus, the reduction in inter-arrival times and batching sizes are based on the view that there would be far fewer passengers entering the station frequently at the same time, as highlighted in the state of the art.

**Table 13:** Comparing inter-arrival times of passengers across three scenarios

Entrance points	Inter-arrival times for Scenarios 0 and 1 (min)	Inter-arrival times for Scenario 2 (min)
Stephenson Street	1	5
Grand Central and Bullring	2	10
Southside and Hill Street	3	15

**Table 14:** Batching in the number of ticket passenger types entering the station: Scenario 2

Entrance points	Batch sizes of passenger entries into the station		
	With advance tickets	Without tickets, towards ticket machines	Without tickets, towards ticket office counters
Stephenson Street	4	1	1
Grand Central and Bullring	3	1	1
Southside and Hill Street	2	1	1

## Results and Evaluation

### Queuing Time Results Across All Scenarios

The queuing times provide useful insight to examine whether there are any blockages in the system across the three scenarios simulated in the same model, with some variations. Since there are queues set up in most activities, the queuing time results are focused on the areas where passengers are likely to form queues, such as at each individual ticket machine, ticket office and ticket gate. The results for all queuing times are presented primarily as table charts, including some graphs to illustrate the findings more visually. As for table charts, the queuing time results only focus on the average and maximum times, as the minimum time for passengers in any queuing system is always zero for this model, where for instance the first passenger using the ticket machine does not have to form a queue. Hence, none of the minimum queuing time results are included, except for the overall results of the simulation model, as discussed in Sect. 7.2.

#### Ticket Machine Queuing Times

Firstly, for those passengers who need to purchase train tickets, they either go towards the ticket machines or go to the ticket office.

Focusing on the ticket machines, passengers may have to form a queue at certain types of ticket machines, where they may use either cash or a card to purchase their tickets. It is anticipated that passengers using cash to buy tickets tend to take slightly longer in interacting with the machine, i.e. carefully inserting notes/coins into the machine where it accepts ‘exact change only’. However, some ticket machines may be out of service due to maintenance or to enable social distancing during the COVID-19 pandemic; this is demonstrated in the scenarios as shown in Sect. 6, and the results of queuing times for ticket machines in Table 15.

**Table 15:** Results of queuing times for all ticket machine types for all three scenarios

Ticket machine no.	Average queuing times (min)			Maximum queuing times (min)		
	Scenario 0	Scenario 1	Scenario 2	Scenario 0	Scenario 1	Scenario 2
Ticket machine type	Card only					
1	0.40	6.44	0.00	2.63	17.31	0.00
2	0.41	6.78	–	3.67	18.96	–
3	0.63	7.41	0.00	3.77	19.70	0.00
4	0.65	7.24	–	4.91	23.74	–
5	0.79	7.79	0.00	4.74	20.48	0.00
6	0.76	–	–	4.69	–	–
7	0.88	–	0.00	4.55	–	0.00
Overall average	0.65	7.13	0.00	4.14	20.04	0.00
Ticket machine type	Cash and card					
1	1.59	11.17	–	8.15	27.24	–
2	1.56	11.90	0.03	7.52	30.78	0.40
3	1.45	11.65	–	6.03	27.57	–
4	1.42	11.33	0.02	7.55	25.77	0.12
5	1.28	11.14	–	7.59	30.87	–
6	0.86	–	0.00	7.11	–	0.00
7	0.83	–	–	5.17	–	–
8	0.76	–	0.00	5.57	–	0.00
Overall average	1.22	11.44	0.01	6.84	28.45	0.13
Overall average (combined)	0.93	9.29	0.01	5.49	24.24	0.07

In Scenario 0, it seems that the majority of passengers are able to obtain their tickets quickly, regardless of the ticket machine types, with an overall average (combined) queuing time of 0.93 minutes. Likewise, in Scenario 2, despite some ticket machines being inactive due to social distancing during the COVID-19 pandemic, passengers using the cash and card type of ticket machines have only waited 0.40 minutes at most. On the other hand, for the card-only type of ticket machine, there are virtually no passengers forming a queue, which suggests that most passengers have already bought their train tickets either online or on their smartphones, and also reflects the decrease in the number of passengers travelling by train during the COVID-19 lockdown. However, in Scenario 1 (excluding the impacts of COVID-19), it is clear that having some ticket machines out of service can cause huge delays of longer queue sizes for passengers to stand and wait at the ticket machines. Hence, this would lead to some passengers missing the train service completely and experiencing further delays, often to their own financial detriment in purchasing another ticket. Therefore, with an excess number of crowds in stations, particularly of commuters during peak hours, it is expected that there would be poorer passenger satisfaction ratings in these circumstances, as indicated from the National Rail Passenger Survey, Transport Focus [28].

#### Ticket Office Queuing Times

For passengers approaching the ticket office, since there are a limited number of ticket office counters, varied across scenarios, the process of purchasing train tickets is typically slower when compared with ticket machines.

In Scenarios 0 and 1, the results of queuing times at ticket office are the same because there is the same number of ticket office counters and the same number of passengers forming a queue. However, when comparing the first two scenarios and the third scenario, in Scenario 2, despite the number of counters being reduced to two from six due to the COVID-19 restrictions of social distancing and the decline in the number of passengers entering the station, the results show that the queuing times at the ticket office are shorter than in the first two scenarios (see Table 16).

**Table 16:** Results of queuing times at the ticket office for all three scenarios

Average queuing times (min)			Maximum queuing times (min)		
Scenario 0	Scenario 1	Scenario 2	Scenario 0	Scenario 1	Scenario 2
3.95	3.95	1.31	15.83	15.83	8.74

Therefore, the results clearly show the contrast between the pre-COVID-19 setting (in Scenarios 0 and 1) and the impacts of COVID-19 lockdown (in Scenario 2) in passengers travelling by rail.

#### Ticket Gate Queuing Times

The queuing times across all scenarios for both sets of ticket gates were consistent, without any major blockages in the system, as seen in Table 17.

**Table 17:** Results of queuing times for both sets of ticket gates for all three scenarios

Ticket gate no.	Average queuing times (min)			Maximum queuing times (min)		
	Scenario 0	Scenario 1	Scenario 2	Scenario 0	Scenario 1	Scenario 2
First set of ticket gates						
1	0.24	0.24	0.08	1.52	1.58	0.62
2	0.23	0.21	–	1.75	1.73	–
3	0.21	0.23	0.07	1.40	1.45	0.46
4	0.21	0.20	–	1.67	1.31	–
5	0.23	0.22	0.09	1.90	1.47	0.54
6	0.20	0.23	–	1.22	1.66	-
Overall average	0.22	0.22	0.08	1.58	1.53	0.54
Second set of ticket gates						
12	0.05	0.04	0.02	0.69	0.61	0.34
13	0.05	0.05	–	0.54	0.60	–
14	0.05	0.05	0.01	0.55	0.71	0.26
15	0.04	0.04	–	0.69	0.63	–
16	0.04	0.05	0.03	0.45	0.68	0.26
Overall average	0.05	0.05	0.02	0.58	0.65	0.29
Overall average (combined)	0.13	0.13	0.05	1.08	1.09	0.41

Between Scenarios 0 and 1, there is a slight discrepancy between the results of all ticket gates; however, with the overall combined average from both sets of ticket gates of its average and maximum queuing times, the results are almost identical. However, in Scenario 2, the key difference is that there are only four ticket gates from both sets of ticket gates that are active to allow social distancing for passengers during the COVID-19 pandemic, when compared with the first two scenarios of 11 ticket gates in total being active.

The results show that despite the reduction in active ticket gates in the simulation, the number of passengers entering through the ticket gates is reduced. Hence, Scenario 2 reflects the findings where the average and maximum queuing times at the ticket gates are more than halved from Scenarios 0 and 1. Therefore, passengers queuing at the ticket gates are unlikely to wait for more than 0.22 minutes, and are likely to wait at most nearly 2 minutes in extreme cases.

### Overall Results of the Simulation Model Across All Scenarios

The overall results of the simulation model, from each scenario, were obtained by examining the end point where passengers exit the simulation. To segregate the results of the ticket passenger types, this is explained in Sect. 5.4 about labels.

As mentioned in Sect. 5.2, simulating 32,494 passenger entries in the model would likely cause the SIMUL8 software program to become unstable and to stop working. Hence, for Scenarios 0 and 1, the number of passengers entering the simulation for this model is roughly around 6000 (nearly 20% of 32,494 entries). However, in contrast to Scenario 2, there are only 574 passenger entries in the simulation (just over 20% of 2632 entries calculated in Sect. 6.3) to reflect the impacts of the COVID-19 pandemic on the decrease in passengers travelling by train. The reason that Scenario 2 simulates 574 passengers rather than 2632 passengers is to keep the number of passengers in the simulation relatively consistent with the first two scenarios to enable a fair comparison.

The overall results of the simulation model for ticket passenger types across all scenarios are listed in Table 18.

**Table 18:** Results of passenger time spent in the simulation for all three scenarios

Scenario 0				
Ticket passenger types	Minimum	Average	Maximum	No. of passengers in the simulation
	Time spent in the simulation (min)			
With advance tickets	6.15	11.81	17.46	3973
Without tickets (ticket machines)	9.31	15.68	25.93	1648
Without tickets (ticket office)	9.16	17.42	33.48	497
All types	6.15	13.31	33.48	6118
Scenario 1				
	Time spent in the simulation (min)			
With advance tickets	6.23	11.80	17.05	3977
Without tickets (ticket machines)	9.26	23.30	46.14	1565
Without tickets (ticket office)	8.87	17.32	31.71	500
All types	6.23	15.24	46.14	6042
Scenario 2				
	Time spent in the simulation (min)			
With advance tickets	6.46	12.02	16.51	401
Without tickets (ticket machines)	9.20	14.87	21.78	88
Without tickets (ticket office)	9.30	15.01	22.59	85
All types	6.46	12.90	22.59	574

Table 19 shows the proportion of passenger time spent in the system within a 15-minute time frame.

**Table 19:** Percentage of passenger time spent within 15 minutes in the simulation for all three scenarios

Scenario no.	Passenger time spent in the simulation within a 15-minute time frame (%)
0	75
1	67
2	80

According to the passenger time spent in the simulation results, the minimum time spent for all types of passengers in the simulation, across all scenarios, was over 6 minutes to reach to the platforms (end point of the simulation).

However, looking specifically at passengers without tickets (ticket machines), there is an increase in time spent from Scenario 0 and 1, where the maximum passenger time spent in the simulation increases from 25.93 to 46.14 minutes. This reflects the difference between having all 15 ticket machines active in Scenario 0, and reducing the ticket machines to 10 in Scenario 1, meaning that some passengers in rare cases would have to wait over 30 minutes in the queue to use the ticket machine. Similarly, for Scenario 2, the ticket machines are reduced to eight to allow social distancing during the COVID-19 pandemic, but unlike Scenarios 0 and 1, the number of passengers entering the station is reduced, suggesting that fewer passengers would be using the ticket machines. Hence, the maximum time spent in the simulation of passengers using the ticket machines in Scenario 2 is decreased to 21.78 minutes from the first two scenarios.

Thus, the changes in the number of active ticket machines, ticket gates and ticket offices to reflect the impact of the COVID-19 pandemic would likely change the behaviour of passengers and crowd distribution in the station. This is because passengers are more likely to buy tickets on their smartphones due to the concern regarding COVID-19 transmission through physical contact with staff at ticket offices and touching surfaces of ticket machines. Therefore, passengers would be less likely to use ticket facilities in the station and would prefer to go directly to the ticket gates, to spend less time inside the station and interacting with staff to minimise the transmission of COVID-19.

### Suggestions for a Better Crowd Management System

The first suggestion is that the time spent for all types of passengers could be improved further to allow them to get through the station towards the platforms in a shorter time. For all scenarios, at least 20% of passengers are taking longer than 15 minutes to reach the platforms. This suggests that there are some blockages in the system such as queues at the ticket machines and ticket office. Hence, for a better crowd management system, passengers could be encouraged to purchase train tickets on their smartphones rather than at the station’s ticket machine or ticket office, to reduce the amount of queuing. Therefore, passengers would be less likely to queue at the ticket machines and ticket office, and would go straight to the ticket gates and then towards the platforms.

A second suggestion could be to remove ticket gates and replace them with sensors which automatically detect passengers through their smartphones. This would help to reduce the passengers’ queuing times in the station and would allow more space for passengers to maintain social distancing during the COVID-19 pandemic. This also means that passengers would not need to go to a ticket office or ticket machine, as the sensors would be linked with a smartphone app where passengers would be automatically charged the correct fare, Loughran [11]. Thus, this would decrease the overall time spent for passengers in the station.

The last suggestion could be to provide a one-way flow system in the main concourse of the station. This is to allow more control in managing the passenger flows, to minimise the risk of overcrowding from passengers entering and exiting the station in the same area. This has already been implemented at the Navigation Street in a separate concourse, where it only allows passengers to exit or change to different platforms. Hence, since there are two sets of ticket gates at the Red Lounge area from the main concourse, the first set of ticket gates near the Stephenson Street entrance could provide a one-way flow system towards the Red Lounge area and then to the platforms, whereas at the other set of ticket gates near Southside and Hill Street, the one-way flow system for passengers exiting the station could be applied as well to allow a more effective crowd management system. This is to avoid conflicting passenger flows, which could lead to overcrowding in some areas of the station. Thus, a one-way flow system could be implemented in Birmingham New Street station, and especially during peak hours and the COVID-19 pandemic, this system would be necessary to provide greater safety and social distancing for passengers using the station, DfT [9].

## Conclusions

Throughout this study, the impacts of rail passenger flow in the Birmingham New Street station concourse prior to and during the COVID-19 pandemic were studied using simulation models implemented in SIMUL8 with three different scenarios. Scenario 0 was set up to analyse the pre-COVID 19 situation and validate the simulation modelling tool. Scenario 1 was set to reflect the changes in passenger behaviour prior to COVID-19, whereas Scenario 2 was developed to analyse the passenger flow in the Birmingham New Street station concourse during COVID-19.

As a result, this study demonstrated that the passenger behaviour at railway passenger stations was changing due to COVID-19. About 75% of the passengers already obtained their tickets prior to entering the station and hence were able to go straight to the ticket gates, meaning that they were also able to avoid queuing at ticket machines or a ticket office. Due to this behavioural change, time spent in the station per passenger on average is now lower than before COVID-19. This made it possible for the number of ticket machines at the station concourse to be reduced by a third, from 15 to 10 machines.

With the COVID-19 impacts on the significant reduction in the number of passengers entering the station, overcrowding inside the station was unlikely to occur, because 80% of all passengers in the simulation had completed their time in the station concourse within a 15-minute time frame. Specifically, passengers were shown to have spent 12.90 minutes on average from entering the station to reaching to the platform levels. This indicated that in the COVID-19 context, passengers were less likely to be waiting in queues for an extended period, being at most 22.59 minutes.

Thus, overcrowding due to passenger queuing inside the station was unlikely during the beginning of the COVID-19 pandemic when there were changes in passenger behaviour in using the station. This led to fewer number of passengers using the station and interacting with ticket facilities to minimise physical interactions with staff and spending less time inside the station to limit the transmission of COVID-19.

As a negative effect, however, due to a reduced number of passengers using the station, the train operating companies were forced to reduce the number of services offered, which resulted in rather limited options for passengers to travel by train. This challenging situation, together with analysis of socio-economic impacts, were not studied in this paper and should be considered for future work.

## Future Work

This study could be expanded by examining other stations in the UK, as well as focusing on the baggage handling systems, i.e. an automatic system using conveyor belts to transfer baggage, to provide a baggage-less journey for passengers, Brice et al. [2]. In addition, it could include a systems design study to introduce a collection point for baggage transfer services at Birmingham New Street; for example, see Yeung and Marinov [31], where such a system is proposed for Newcastle Central.

With regard to the simulation model itself, it could be improved by widening the scope of the system, as this model is primarily based around the main concourse and the Red Lounge area, and the platform levels serving as an end point of the simulation. Hence, this model would need to provide more development and attention at the platform levels because it is likely that crowds would start to emerge onto the platforms as they wait to board the train. However, this could lead to overcrowding and bottlenecks of passengers accessing the platforms, often via escalators. This poses a significant risk of passenger safety because, as Kabalan et al. [10] noted, very high crowd density on the escalators could lead to incidents of trips and falls. Additionally, at platforms, heavy crowds could lead to someone falling onto the tracks, potentially leading to a fatal accident. Therefore, this provides an opportunity to examine the effectiveness of crowd management at the platform levels by using simulation models and scenarios to propose strategies to ensure safety for all passengers.

Another opportunity to examine the effectiveness of crowd management is through the use of technology. As suggested by Al-Shaery et al. [1], during public events and mass gatherings, crowd detection and monitoring techniques could be used to control and track the crowd behaviour in order to mitigate the risk of incidents and tragedies. This is achieved through the use of automated technology tools such as radio-frequency identification (RFID), Wi-Fi and Bluetooth to detect and monitor the crowd. Likewise, in the study by Yang and Lam [30], with the development of smart cities such as in Hong Kong, crowd management systems have become more widespread in detecting and tracking crowds in real time. This is done through monitoring of crowd dynamics and density using information and communications technology (ICT) such as closed-circuit television (CCTV) cameras, video analytics and information dissemination systems.

Thus, both studies, using technology to investigate crowd management, could be implemented in this study in a railway station context. For example, RFID could be applied for physical train season tickets purchased by passengers who regularly commute by train, which would be useful in detecting and tracking passengers to provide real-time travel information as they enter the station. For passengers who have purchased train tickets on their own smartphones, the use of Wi-Fi or Bluetooth could be used, similarly to RFID, to detect and track passengers as they enter the station. Also, CCTV cameras could be used to monitor passengers in the station at a wider scale and much more cheaply than with other automated technology tools to analyse crowd management. Therefore, the rationale of using technology to examine crowd management is to minimise the risks associated with overcrowding and to provide safety for all passengers using the station. However, it is important to consider that automated technology tools are innovative and can be expensive initially when implemented. Nevertheless, the use of technology is useful in understanding crowd dynamics, density and behaviour in use of the station.
